# Comparison of analog and digital patient decision aids for the treatment of depression: a scoping review

**DOI:** 10.3389/fdgth.2023.1208889

**Published:** 2023-09-01

**Authors:** Jana Sedlakova, Anna Lisa Westermair, Nikola Biller-Andorno, Christoph A. Meier, Manuel Trachsel

**Affiliations:** ^1^ Institute of Biomedical Ethics and History of Medicine, University of Zurich (UZH), Zürich, Switzerland; ^2^ Clinical Ethics Unit, University Hospital of Basel (USB), Basel, Switzerland; ^3^ Clinical Ethics Unit, University Psychiatric Clinics Basel (UPK), Basel, Switzerland; ^4^ Department of Internal Medicine, University Hospital Zurich (USZ), Zürich, Switzerland; ^5^ Medical Faculty, University of Geneva, Geneva, Switzerland; ^6^ Medical Faculty, University of Basel, Basel, Switzerland

**Keywords:** patient decision aids, shared decision making, autonomy, depression, mental health, digitalization

## Abstract

**Introduction:**

Patient decision aids (PDAs) are important tools to empower patients and integrate their preferences and values in the decision-making process. Even though patients with mental health problems have a strong interest in being more involved in decision making about their treatment, research has mainly focused on PDAs for somatic conditions. In this scoping review, we focus on patients suffering from depression and the role of PDAs for this patient group. The review offers an overview of digital and analog PDAs, their advantages and disadvantages as well as recommendations for further research and development.

**Methods:**

A systematic search of the existing literature guided by the Cochrane Handbook for Systematic Reviews and the Preferred Reporting Items for Systematic Reviews and Meta-Analyses - extension for scoping reviews (PRISMA-ScR) was conducted. Three electronic literature databases with the appropriate thematic focus were searched (PubMed, PsycInfo, and Web of Science). The search strategy used controlled and natural language to search for the key concepts decision aids and depression. The articles were selected in a two-step process guided by predefined inclusion and exclusion criteria. We narratively synthetized information extracted from 40 research articles.

**Results:**

We included 40 articles in our review. Our review revealed that there is more focus on digital PDAs in research than in clinical practice. Digitalization can enhance the benefits of PDAs by developing tools that are more efficient, interactive, and personalized. The main disadvantages of both types of PDAs for the treatment of depression are related to time, dissemination, and capacity building for the health care providers. Digital PDAs need to be regularly updated, effective strategies for their dissemination and acceptance need to be identified, and clinicians need sufficient training on how to use digital PDAs. There is more research needed to study which forms of PDAs are most appropriate for various patient groups (e.g., older adults, or patients with comorbidities), and to identify the most effective ways of PDAs' integration in the clinical workflow. The findings from our review could be well aligned with the International Patient Decision Aids Standards.

**Discussion:**

More research is needed regarding effective strategies for the implementation of digital PDAs into the clinical workflow, ethical issues raised by the digital format, and opportunities of tailoring PDAs for diverse patient groups.

## 1. Introduction

According to the World Health Organization (WHO), about 280 million people worldwide suffer from a depressive disorder rendering it a leading cause of disability in the world (1). Depression as a public health issue becomes even more challenging when we consider therapeutic options, such as different types of psychotherapy and psychiatric medication (2, 3). Even though there are effective, evidence-based treatments, only about one third of patients receive adequate therapy (4, 5). Some of the reasons for this problem are poor clinician-patient communication, insufficient knowledge of treatment options as well as insufficient inclusion of patients' views and preferences into the decision-making process (6–8). Therefore, the better inclusion of patient values and preferences into treatment decisions may play an important role in addressing the public health issue of depression (9, 10) by possibly enhancing the quality of treatment outcomes, through enhanced patient satisfaction and therapeutic adherence (11). Furthermore, the importance of involving patients and their values and preferences is consistent with best practices and clinical guidelines for mental health services (9, 12–14).

Patient decision aids (PDAs) are important tools for facilitating and achieving this aim. PDAs are evidence-based tools that inform patients about treatment options and go beyond mere informational materials by helping patients elicit their preferences, and thus preparing them for a consultation with a healthcare professional to engage in a shared decision making (SDM) process (15–17). PDAs aim at empowering patients by presenting the available evidence in an understandable manner, thereby encouraging the patients to be more involved in the decision making, reducing their decisional conflict, and aligning treatment decisions with patients' preferences and values (15, 16, 18, 19). These are important factors both for patient's empowerment and SDM that is based not only on information exchange but also on creation of a trusting relationship between a mental health care provider and a patient (17).

PDAs can be designed as stand-alone tools, as facilitators during the SDM process, or as a combination of both. There are several types of PDAs: they can be either developed in analog forms, e.g., as fact sheets, or in a digital format, such as websites or applications. Best practice standards for developing PDAs have been defined in the International Patient Decision Aid Standards (20, 21).

As in many other areas, novel IT technologies hold many promises also in the health care sector. This does not only apply to diagnostic tools or treatment options but also to decision making. Digitalization offers the opportunity to design PDAs in an interactive, personalized, and possibly more effective way to better engage patients and facilitate SDM for decisions regarding current and future care (22–24). Hence, digital PDAs have the potential to empower patients and orient healthcare towards patient- and value-oriented practice (10, 22, 24).

Previous research on PDAs has mainly focused on somatic conditions (18, 25, 26). A Cochrane review (18) showed the effectiveness of PDAs in terms of increased patient knowledge, decreased decisional conflict, and clarity about personal values. However, the majority of the 105 included studies in the review focused on health decisions related to somatic conditions (18). A similar pattern can be seen in a recent review focusing on users' involvement in design and development of PDAs; also here, only about 2% of the included PDA projects belong to mental health (27). These reviews are used to formulate guidelines and checklists for development of PDAs (28, 29), but the patient groups with major depressive disorder and generally with mental disorders are underrepresented. The inclusion of patients and their preferences and values in the decision making process is at least as important in mental healthcare. Nevertheless, PDAs have only recently been researched in mental healthcare, even though patients with mental disorders in general and depression in particular are interested in taking a more active role in decision making (30, 31). The (qualitative) research specifically focusing on patients' experiences with PDAs and their involvement in the development of PDAs for the treatment of depression deserves more attention (30, 32). Furthermore, recent reviews have focused on quantitative results of randomized-controlled studies, thus neglecting a large part of the available evidence (33, 34). Current research on SDM in mental health care can provide valuable insights for future studies on PDAs as it offers a crucial context for understanding their utilization (11, 17, 35). Additionally, such research highlights important factors that contribute to the empowerment and active involvement of patients with mental health conditions in their treatment (17, 35).

To follow the current development driven by digital revolution and its potential, the aim of this scoping review was to characterize and compare digital and analog PDAs for patients with major depressive disorders by portraying both qualitative and quantitative evidence of their main advantages and disadvantages. Thereby, we define digital PDAs as tools that can be used with computers, mobile devices, or other digital devices. Analog PDAs are tools that are not in digital electronic formats. Instead, they typically use paper-based materials, such as flyers or booklets. This evidence synthesis provides a comprehensive understanding of the role of PDAs for patients' decision making as well as recommendations for further research and development.

## 2. Methods

**Figure 1:**
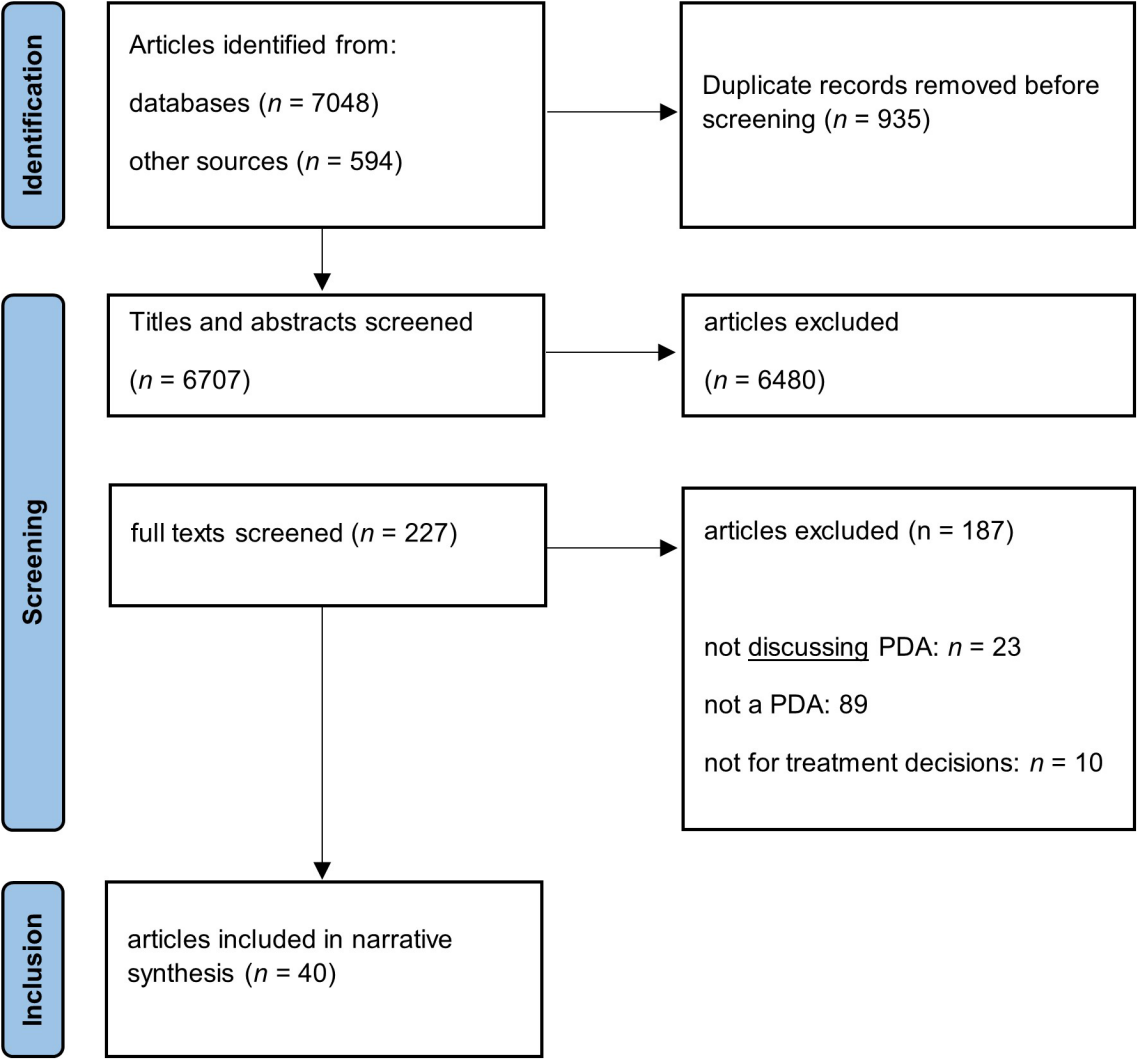
PRISMA flowchart of the literature search. MDE, major depressive episode, PDA, patient decision aid.

As we intended to portray the existing literature on key characteristics of PDAs in depression (rather than provide a definitive, quantitative answer to a narrow question such as the effect of PDAs on decisional conflict in patients with depression), scoping review methodology was most appropriate for our study (36). As standard registries such as PROSPERO do not currently accept Scoping Reviews we did not pre-register the review (37). A systematic search of the existing literature guided by the Cochrane Handbook for Systematic Reviews (38) and the Preferred Reporting Items for Systematic Reviews and Meta-Analyses—extension for scoping reviews (PRISMA-ScR) was conducted (see the PRISMA Flow Diagram in Figure 1) (39). Three electronic literature databases with the appropriate thematic focus were searched (PubMed, PsycInfo, and Web of Science). The search strategy used controlled and natural language to search for the key concepts decision aids and depression (see Supplementary Table S1). The database search closed on December 31st, 2022. To compensate for eventual shortcomings of the database search, it was complemented with a search on Google Scholar, and, for all included articles, a search for citing articles on Web of Science and a hand search of the reference lists.

The articles were selected in a two-step process guided by predefined inclusion and exclusion criteria (see Table 1). To broaden the scope of our review, we did not restrict our search by publication date and included a variety of article types such as study protocols and reviews. Screening was done independently by ALW and JS. In both screening steps, ALW screened all records, while JS screened 20% including all references marked as “unsure” by ALW and a random selection of references. Disagreements were resolved by discussion among ALW, JS, and, if necessary, MT.

**Table 1:** Inclusion and exclusion criteria.

Included in this review were articles	Excluded from this review were articles
1. Discussing a) positive and/or negative aspects, (dis)advantages, benefits, or risks of or b) stakeholders’ attitudes and opinions towards	1. Mentioning, but not discussing PDA for MDE such as a) reviews that do not contain more information on PDA for MDE than is cited from the primary studies, such as (93) b) published PDA for MDE without any form of evaluation, such as (94)
2. Patient decision aids (PDA)—defined as a) objects, such as worksheets, booklets, apps, comics, videos, etc. b) designed for use by patients (together with a mental health professional or before/after a consultation—thus including encounter DA) as evidenced by, e.g., the use of easy, lay language, tailoring information to the individual, and/or visualization of numerical information, c) informing about several options and providing interventions to support patients’ decision-making process (such as pro-con-lists or preference elicitation tasks), d) based on a neutral and balanced aggregation of scientific evidence, and e) aimed at an optimal decision process, not at a specific decisional outcome—for	2. Not concerned with PDA, but rather a) decision support in the form of human interaction without using any tool, such as peer decision support b) aids not primarily intended for use by patients, such as communication aids or pharmacogenetic decision aids (95) c) mere information materials, such as (96) d) advertisement from pharmaceutical companies, such as (97), or aggregated data from online fora, such as (98) e) algorithm-derived treatment recommendations, such as (99)
3. Decisions about type and/or duration of treatment of	3. Not concerned with decisions about type and/or duration treatment of MDE, such as a) tools encouraging persons with depressive symptoms to access mental health care (100) b) generic decision aids (101)
4. Unipolar major depressive episodes (MDE) diagnosed by a mental health professional	4. Concerned with other conditions, such as self-reported depressive symptoms, positive depression screenings, dysthymia, organic affective disorders, or bipolar depression (102)
5. Published in a peer-reviewed journal listed in the Index Medicus (including reviews, opinion articles, editorials)	5. That are a) conference abstracts, such as (103) b) previous versions of reviews for which an updated version has been published, such as (104)
6. Written in English or German	6. Whose full text was not ascertainable, such as (105)

Data extraction was aligned with the aims of the study: summarizing advantages and disadvantages of using PDAs in general and digital vs. analog PDAs. During the extraction process, it became clear that it was not possible to differentiate between benefits and advantages or risks, challenges, and disadvantages, because the included articles did not differentiate clearly between this terminology. Besides, the terminology used in the selected articles was not unified. That is why we decided to use general terms of advantages and disadvantages to refer to the potential positive and negative aspects of the use of digital and analog PDAs. The method of narrative synthesis (40) was chosen to gather a broad scope of knowledge to create an overview of PDAs for depression and guide further research. This method is well suitable since there are not many studies about PDAs for depression and given the variety of the chosen articles. To compare the digital and analog PDAs and provide an overview on the digital PDAs, the information extraction was clustered in accordance with the topics of advantages, disadvantages, and recommendations for both digital and analog PDAs. Data extraction was done by JS and checked by ALW.

This narrative synthesis was supplemented by tabulating the quantitative results. For ease of comparison across publications, effect sizes are presented wherever possible. For included studies reporting neither effect sizes nor sufficient information to calculate them although this should be possible given the study design, the corresponding authors were contacted with a request for additional information. Cohen's *d* was chosen because it is appropriately widely used for continuous outcomes (such as decisional conflicts) independent of the scale on which the outcome was measured (41). Cohen's *d* is computed as the difference of the two means divided by the pooled SD with values of 0.20, 0.50, and 0.80 signifying small, medium, and large effects, respectively (42). For publications not reporting Cohen's *d*, it was computed as follows: when raw data or descriptive statistics such as M and SD were reported, *d* was derived from these statistics. From CIs, the SD was computed as proposed by Higgins (43). From *F* statistics with one degree of freedom from *t* statistics, and from odds ratios, *d* was computed according to Borenstein (2019) (41), and from *z* and *χ*^2^ statistics according to Rosenthal and DiMatteo (44). For pre-post-control between-subject designs, *d* was computed according to Morris (45).

## 3. Results

After two-step screening and applying our inclusion and exclusion criteria, 40 articles remained (see in Figure 1) that were used to synthetize evidence and information on disadvantages and advantages of the use of analog and digital PDAs as well as recommendations for their development. Table 2 shows the main characteristics of the 40 included publications for this review (15, 25, 26, 30–33, 46–78). More than one third of the articles focused on digital PDAs for patients with depression without any focus on a particular patient group (e.g., older adults or young adults). Very often, the articles included not only the patients' but also the clinicians' perspective on PDAs. Most PDAs were developed in interdisciplinary teams and in line with the International Patient Decision Aid Standards (61, 69, 70, 72).

**Table 2:** Main characteristics of the included articles (*n* = 40).

First author	Year	Language (s)	Decision	Type of PDA	Target group	Context of use	Stakeholders included
Abousheishaa et al. (46)	2022	E	Use of antidepressants	Analog		During mental health care encounter	Patients, psychiatrists, experts in shared-decision making in mental health
Alarcon-Ruiz et al. (33)	2022		Between forms of treatment	PDAs in general	Adults		
Aljumah et al. (47)	2015	A	Use of antidepressants	Analog (booklet)	Adults (18–60 years old), newly diagnosed	Pharmacy visit for antidepressants	Unspecified experts
Aoki et al. (48)	2022	E	Discontinuing antidepressants	Analog (booklets)	Patients having achieved remission with monotherapy	Independently of health care encounters	Patients, health care providers, experts on depression and decision aids
Aoki et al. (49)	2019	J	Between forms of management	Analog (booklet)	University students with first episode	Between health care encounters, alone and during nurse encounter	-
Barr et al. (50)	2019	E	Between forms of management	Digital, tablet-based, static	Primary care patients	Before and during primary care encounter	Members of the public, Researchers, patients, caregivers, medical assistants, clinicians, depression experts
Beaulac et al. (51)	2016	E, F	Between forms of treatment when considering initiation, change, or (dis-)continuation	Digital, web-based and brochure version	Primary care patients	Independently of or before, during, or after a health care entcounter	Mental health professionals, young adults
Brodney et al. (52)	2021	E	Between forms of management	Electronic (DVD) and analog (booklet)	Adults		
Broughton et al. (53)	2021		Antidepressant use during pregnancy	PDAs in genereal	Women, pregnant or planning a pregnancy		
Dannenberg et al. (54)	2019	E	Between forms of management	Digital, tablet-based, interactive		In the waiting room before the primary care encounter	Researchers, patients, and primary care providers
Fisher et al. (55)	2021	E	Between forms of management	PDA in preparation	Patients with problematic alcohol use		Patients, family members, mental health care providers
Gordon et al. (56)	2016	E	Between forms of treatment	Digital, tablet-based, with personified interface	Low income, ethnic/racial minority pregnant women	In the waiting room before the clinical encounter	Low-income women with history of depression in pregnancy, prenatal care providers, administrators, mental health services researchers, an application developer
Hetrick et al. (30)	2008		Use of SSRI	PDAs in general	Children and adolescents		
Hopwood et al. (25)	2020		Use of antidepressants	PDAs in general			
Hussain-Shamsy et al. (57)	2022	E	Start or continue antidepressants during pregnancy	Digital, web-based, interactive	Adult women, pregnant or planning a pregnancy	Adjunct to, but for use outside of clinical care	Medical experts, members of the community, end users
Kivelitz et al. (59)	2018		About treatment setting	PDA in preparation	Adults		Patients
Kroenke (60)	2015		Between forms of treatment	PDAs in general			
LeBlanc et al. (61)	2013	E	Between antidepressants	Analog (laminated cards, leaflet)	Adults	During the primary care encounter	Patients, clinicians, policy makers
LeBlanc et al. (62)	2015						
Loh et al. (63)	2007	G	Between forms of treatment	Analog (decision board)	Patients with depression in primary care setting	During primary care encounters	
Loh et al. (64)	2007						
Perestelo-Perez et al. (32)	2017	S	Between forms of treatment	Digital, web-based		Between primary care encounters	Patients, health professionals
Raue et al. (31)	2010	E, S	Between forms of management	Analog (one-page form)	Elderly (65+ years old) minority primary care patients	During primary care nurse encounter	-
Raue et al. (66)	2011						
Raue et al. (65)	2019						
Reis (67)	2021	E	Between forms of treatment	Digital, web-based, interactive		Participant-initiated, unrelated to health care encounters	
Reuter et al. (68)	2022	E	Between forms of management	Digital, web-based application (additional paper copy)	Patients with coronary heart disease	In the waiting room before the primary care/cardiologist encounter	Patients, primary care providers, cardiologists, mental health care providers, administrators, developers, experts in user experience, behavior change, and patient activation
Rogojanski et al. (69)	2020	E, F	Between forms of treatment	Digital, web-based, static	College students	After a health care encounter	Researchers with backgrounds in psychology, psychiatry, pharmacy, and knowledge mobilization; health professionals
Shillington et al. (70)	2020	E	Between forms of augmentation	Digital, web-based, interactive	Adults with treatment resistant depression	In preparation of and during a mental health care encounter	Patients, a patient advocate, mental health professionals, researchers, an expert in shared decision making
Simmons et al. (26)	2011		Any decision regarding treatment	PDAs in general	Adolescents and young adults (12–24 years old)		Adolescents and young adults (12–24 years old), their caregivers
Simmons et al. (71)	2013		Any decision regarding treatment	PDAs in general	Adolescents (12–18 years old)		Health professionals
Simmons et al. (72)	2017	E	Between forms of management	Digital, website presented on tablet	Adolescents and young adults (12–25 years old)	In enhanced primary care encounter	Patients, caregivers, clinicians, experts in youth depression, shared decision making, and biostatistics
Simon et al. (73)	2012	G	Between forms of treatment	Digital, Web-based, interactive, tailored to the individual	Adults insured by a specific health insurance	Participant-initiated, unrelated to health care encounters	Tested by patients and health care providers
Weiss et al. (78)	2010						
Stacey et al. (15)	2008		Between forms of treatment	PDAs in general		Adults	Patients
Starks et al. (74)	2015	E	Depression management	Digital, tablet-based, interactive	Alaskan Native and American Indian people	Short version for use during a primary care encounter, and more comprehensive version for use outside of the encounter	Tribal health system leaders, the Indian Health Service Alaska Area Institutional Review Board and tribal research review committees, project steering committee, healthcare providers, software contracting firm, customer-owners, researchers
Vigod et al. (75)	2016	E	Use of antidepressants during pregnancy	Digital, web-based, interactive	Adult women, pregnant or planning a pregnancy	In addition to, but for use outside of clinical care in a specialist or non-specialist setting	Perinatal psychiatry experts, perinatal mental health providers, patient decision aids experts and a health care technology company
Vigod et al. (76)	2016						
Vigod et al. (77)	2019						
Khalifeh et al. (58)	2019						

Language(s) of the PDA: A, Arabic; E, English; F, French; G, German; J, Japanese; S, Spanish. PDA, patient decision aid; SSRI, selective serotonin reuptake inhibitor. Articles on the same PDA are group together.

### 3.1. Advantages

#### 3.1.1. Advantages of both digital and analog PDAs

The mentioned advantages of PDAs and positive roles that PDAs can have for patients seeking treatment of depression were increased knowledge (25, 32, 46, 48, 50, 51, 54, 58, 62, 69, 70, 73, 75, 77), reduced decisional conflict (32, 55, 58, 62, 70, 72, 73, 77, 78), supporting decision-making (56, 62, 72, 76), elicitation of and treatment alignment with patients’ preferences and values (26, 32, 46, 51, 54, 72, 76, 77), better preparation for and involvement in SDM (15, 26, 46, 48–51, 55, 63, 64, 69, 71, 72, 78), patients' satisfaction (46, 49, 51, 54, 62–64, 72), and more realistic expectations (51, 72). Furthermore, some studies pointed out that the use of PDAs in SDM did not increase the consultation time (25, 46, 49, 62, 63). Finally, an inclusion of personal stories was considered to be beneficial for patients' elicitation of their preferences because they could relate to people with similar experiences (48, 59, 68).

Supplementary Table S2 displays an overview of quantitative results from the included studies. These provide preliminary evidence for good acceptability, a reduction of decisional conflict, increase of patient involvement, adherence, and satisfaction by PDAs, without increase in consultation time. Data on other outcomes such as patient knowledge and clinical outcomes are inconsistent and/or scarce.

#### 3.1.2. Advantages specific to digital PDAs

A variety of advantages or positive aspects specific to digital PDAs for the treatment of depression were discussed: digital PDAs are effective, easy and quick to use and access (50, 51, 54, 67). They can give patients enough flexibility and time to use them when it is most suitable and comfortable for them without being rushed (54, 55, 75, 77). This should enable patients to be better prepared for the consultation with clinicians, formulate questions, or use the waiting time efficiently if the tools are used in the waiting room (54, 55). Furthermore, audio and visual components can be implemented in digital PDAs that can be particularly important for low literacy users (56). Digital PDAs can also offer more privacy, for example, when the tool is secured with a password (51, 75). They can be important for particular groups such as young patients as they are comfortable with this technology (67, 71, 72). Furthermore, digitalization allows for personalization and tailoring of PDAs (15, 30, 50, 69). Digitalization also allows greater scalability and adaptability of the PDAs in terms of both content (e.g., updates in the light of new evidence or personalized content) and form (e.g., information online or in printable format) (50, 57, 75, 76). The scalability and adaptability can lead to further implementation of PDAs in other countries (57). More specific advantages, which are mentioned, were the possibility to include exercises that will prepare patients for decision making and help them understand how their decision making is influenced by relatives or friends (57, 75).

Finally, from the clinicians’ perspective, a great advantage of digital PDAs used in SDM settings is the possibility to link them with electronic health systems, e.g., electronic health records or screening assessment tools (56). This will create better efficiency and allow real-time decisions (50, 54). Integration of digital PDAs can also lead to support evidence-based and patient-centered care (54, 72).

#### 3.1.3. Advantages specific to analog PDAs

The only mentioned advantages specific to analog PDAs for the treatment of depression were that printed materials are important for patients without access to computers or the internet (46, 51), and that they are freely available from public and non-profit organizations (71). An overview of all advantages is in Table 3.

**Table 3:** Advantages of PDAs.

Digital	Analog	Both
Effective, easy and quick to use and access (50, 51, 54, 67)	For patients without access to computers or internet (46, 51)	Increased knowledge (25, 32, 46, 48, 50, 51, 54, 58, 62, 69, 70, 73, 75, 77)
Flexibility and sufficient time for the usage (54, 55, 75, 77)	Free available from public and non-profit organizations (71)	Reduced decisional conflict (32, 55, 58, 62, 70, 72, 73, 77, 78)
Inclusion of video and audio materials (56)		Supporting decision-making (56, 62, 72, 76)
More privacy (51, 75)		Elicitation of and treatment alignment with patients’ preferences and values (26, 32, 46, 51, 54, 72, 76, 77)
More suitable for particular groups with high affinity for technology (67, 71, 72)		Not increased consultation time (25, 46, 49, 62, 63)
Personalization (15, 30, 50, 69)		Better preparation for and involvement in SDM (15, 26, 46, 48–51, 55, 63, 64, 69, 71, 72, 78)
Greater scalability and adaptability (50, 57, 75, 76)		Patients’ satisfaction (46, 49, 51, 54, 62–64, 72)
Inclusion of exercises and involvement of family and friends (57, 75)		More realistic expectations (51, 72)
Better efficiency and real-time decisions (50, 54)		
Linkage with electronic health systems (56)		
Support of evidence-based and patient-centered care (54, 72)		

### 3.2. Disadvantages

#### 3.2.1. Disadvantages related to both digital and analog PDAs

The challenges, which were discussed either in the general context of PDAs or for both digital and analog PDAs for the treatment of depression, were mainly related to the information provided in the PDAs. Both clinicians and patients raised concerns about PDAs that appeared to be too technical and too overwhelming, for example, in terms of content or wording that they provide (32, 48, 50, 51, 68). However, some patients and clinicians also reported that the material was insufficient (49, 50) or the presentation of treatment was conflicting information provided by a clinician (58). Patients' literacy needed for using PDAs that might present a challenging issue (50, 68). Finally, the use of PDAs might pose an additional burden for patients (58, 59) and increase their anxiety (58).

#### 3.2.2. Disadvantages specific to digital PDAs

The common challenges discussed with digital PDAs for the treatment of depression were mainly connected with PDAs integration and implementation in the clinicians' workflow (50, 51). PDAs can play an important role to support SDM and hence, clinicians need sufficient training on how to properly integrate PDAs into their consultation (50). Another problematic issue might be the dissemination of PDAs (51). Finally, digital PDAs might not be suitable and easy to use for all groups of patients (54).

#### 3.2.3. Specific to analog PDAs

In analog PDAs for the treatment of depression, it might be particularly challenging to regularly update evidence about the treatment (26). This challenge is intensified considering scarce resources—such as finances—for creating analog materials (26). Table 4 displays an overview of all disadvantages.

**Table 4:** Disadvantages of PDAs.

Digital	Analog	Both
PDAs integration and implementation in the clinicians’ workflow (50, 51)	Challenging regular updates of evidence about the treatment (26)	Appropriate amount and form of information provided (32, 48, 50, 58, 69)
Resources for trainings for clinicians on how to integrate PDAs (50)	Not enough resources such as finances for the creation of PDAs (25)	Patient literacy (50, 68)
Dissemination of PDAs (51)		Usage can be perceived as additional burden (58, 59)
Not suitable for all patient groups (54)		

### 3.3. Recommendations

#### 3.3.1. Recommendations for both digital and analog PDAs

In the papers included in this analysis, several recommendations have been identified, which relate to both analog and digital PDAs for the treatment of depression (Table 5 display an overview of recommendations and their relevance) and correspond with the International Patient Decision Aids Standards: information in PDAs should be written in clear, understandable, concise, and simple language (46, 50, 51, 54, 57). Furthermore, careful wording about potential risks should be used (54, 70) as well as balancing them with positive effects (46). It was recommended that clear instructions or even education should be provided on how to use PDAs (48), particularly digital PDAs (54). Specific recommendations about the visual side of PDAs included recommendation for bright and attractive colors (46, 50), and the use of more visuals for risks and expected benefits of treatment options (46, 70). The inclusion of important stakeholders for designing and developing PDAs was also recommended (55, 58).

**Table 5:** Recommendations for future research.

Research focus	Relevance
Inclusion of a variety of patient groups (32, 51, 62, 70, 72)	Determination of effectiveness for a broad population. Identification of PDAs’ accessibility for different health literacy levels. Enabling appropriate personalization of PDAs.
Identification of the precise role of PDAs in SDM and the optimal amount of information provided in PDAs (33, 48, 69, 72)	Important for PDAs acceptance, development, design and inclusion in the clinical workflow.
Implementation of PDAs (33, 46, 58, 62, 67, 68, 77)	Important for PDAs acceptance, development, design and inclusion in the clinical workflow as well as cost-effectiveness considerations.
Replication of studies (33, 48, 69, 72)	More robust evidence about the effectiveness of PDAs.

In terms of the content of PDAs for the treatment of depression, it was suggested to include both pharmacological as well as psychotherapeutic treatment options (69), and to include a broad range of questions regarding possible treatments (51), update the content regularly (e.g., every 2 years) or, alternatively, determine the “expiration date” of PDAs (50). One study recommended to consider a bias possibly inflicted by the order for which treatment options are presented (69). Furthermore, the inclusion of patients' values and preferences as well as personalized information based on their current social situation, religious and cultural beliefs, and prior knowledge was highlighted several times (26, 31, 74). This can be expected to encourage patients to raise issues, which are important for them and which they would not raise otherwise in the consultation with a clinician (26).

Tailoring PDAs to the needs of the target group was another recurring topic (31, 33, 51, 60). In the context of older adults, it was recommended to tailor PDAs for the treatment of depression in a way that the influence of cognitive impairment is minimized (31). Several studies highlighted the importance of designing PDAs for the treatment of depression for different subgroups of patients (51), such as specific age groups, ethnicities, educational level, and patients with medical comorbidities and other disabilities (31, 62, 70).

In terms of research, it was also recommended to include a variety of population groups as well as considerations of factors related to age and involvement of caregivers (72). Future research should replicate existing studies and focus in more detail on the extent to which PDAs are effective for the treatment of depression and adherence to treatment, and whether PDAs have uniquely positive effects on SDM (33, 48, 69, 72). Furthermore, more research is needed to determine the precise role of PDAs in SDM, to identify the optimal amount of information provided in PDAs (69), and PDAs' accessibility for different health literacy levels (49, 50). Finally, it was recommended to conduct more research on implementation of PDAs (77), more specifically on facilitators and barriers for implementing PDAs (33, 46, 67, 68) as well as on cost-effectiveness of implementing and developing PDAs (58, 62).

In terms of use of PDAs for the treatment of depression, it was recommended to make PDAs interactive, use them on a regular basis (26), make them available at an early stage of decision making (69), include healthcare professionals such as clinicians or nurses to use PDAs with patients (31, 74), and to also include caregivers and relatives (26, 48). Particularly in the context of the last point, it was recommended to provide patients with PDAs before a consultation so that family and other important people for patients can be included in the decision process (49).

#### 3.3.2. Recommendations specific to digital PDAs

Recommendations specific to digital PDAs for the treatment of depression mainly concerned their implementation in the clinical workflow (50, 51, 54, 68): PDAs could be delivered and accessed by patients directly at the clinic by using electronic tablets (50, 68). If linked with screening assessment, the waiting time could be used effectively, and this would allow real-time decision support (54). From a clinicians' perspective, it was recommended to implement PDAs into electronic medical record systems and make them accessible within a shared network (e.g., electronic charts) (50, 54). More research on how exactly such implementation can be reached is needed (54, 68). Finally, it was recommended to further study what forms of digital PDAs (e.g., mobile applications or websites) are most powerful for both patients and healthcare providers (51).

## 4. Discussion

This scoping review aimed at comparing digital and analog PDAs for patients with major depressive disorder by collecting evidence and information regarding advantages and disadvantages of their use as well as recommendations for their development. The main finding was that analog and digital PDAs increased the patients' satisfaction with the tool, enhanced knowledge, reduced decisional conflict and better preparation for SDM. There was only one advantage specific to the analog PDAs, namely that these PDAs are more suitable for people without access to technology such as computers or smartphones. Digital PDAs were deemed to be more efficient, flexible, more easily accessible and with the opportunity of personalization. The main disadvantages of both types of PDAs for the treatment of depression are related to time, dissemination, and capacity building for the health care providers. Digital PDAs need to be regularly updated, effective strategies for their dissemination and acceptance need to be identified, and clinicians need sufficient training on how to use digital PDAs. Furthermore, there is more research needed to study which forms of PDAs are most appropriate for various patient groups (e.g., older adults, or patients with comorbidities), and to identify the most effective ways of PDAs' integration in the clinical workflow.

The findings from our review could be well aligned with the International Patient Decision Aids Standards (20, 21) that was often used as guidance in the development of PDAs. In both, there is a strong emphasis on presenting treatment options based on evidence that is regularly updated, presenting options in understandable language, including patients' values, and developing them in a way that can guide patients in SDM. The specific topic that was not discussed in our findings is the presentation of probabilities of outcomes. Our findings offer additional insights regarding tailoring PDAs to different patients' subgroups, need for specifying dissemination and implementation process of PDAs and inclusion of the role of families and significant persons in the decision making.

The review of the literature documents the growing interest in digital PDAs for patients with depression. These patients deem it important to be more involved in the decision-making process and have more information about the available treatment options (25, 26, 32). Digitalization offers greater scalability, flexibility, and personalization of PDAs, which would allow for a possibly more effective and tailored inclusion of patients' values and preferences into the decisional framework for therapeutic choices. The easy access, flexibility and personalization of digital PDAs might be especially beneficial considering patients' possible cognitive deficits such as lower motivation or poor concentration. Furthermore, an advantage of digital PDAs for the treatment of depression is its effective inclusion in the clinical workflow, which should facilitate evidence-based and patient-centered healthcare.

### 4.1. Research gaps and recommendations for further research

In general, more research is needed to systematically study the clinical effectiveness and possibly adverse effects of digital PDAs for the treatment of depression. The present review shows a high level of heterogeneity of approaches and measures of digital PDAs. Some studies were designed with a narrow focus on a specific patient group; other studies had a broad focus on PDAs from both clinicians' and patients' perspective. In addition, many studies used unvalidated instruments to measure the impact of the intervention, such as treatment adherence, patient knowledge, and goal concordance of care. This is in part explained by a lack of validated measurement instruments, e.g., for concordance of care with patient preferences and values (32). Another challenge for interpreting the existing evidence is the different study designs that ranged from purely descriptive studies and pilot testing in focus groups with baseline measures to randomized control trials (RCTs). This is in line with other recent recommendations for further research on decision aids, which call for more RCTs in this field using checklists to ensure that all relevant factors are measured (79).

In the present scoping review, only 40 articles qualified for inclusion, although inclusion criteria were deliberately broad. This is a small number given the impact of depression on public health as well as on patients, who often wish to be included to a greater extent in the decision-making process about their treatment. This research gap is accentuated by the fact that half of the articles had to be excluded because the main (or the only) focus was on the use of decision aids by clinicians (see Figure 1). Even though clinicians' experience can offer valuable insight particularly regarding the role of PDAs in SDM (60), a stronger focus of future research should be on patients' experience. Interestingly, among the articles after the first screening step, PDAs powered by artificial intelligence (AI) were mainly developed for diagnostic or screening purposes (80, 81) even though the potential of AI could be used to contribute to personalized and tailored PDAs as well.

The development, challenges and research of PDAs for patients with depression can gain from taking inspiration from research on PDAs for somatic conditions such as osteoarthritis and cancer and for other mental disorders such as schizophrenia (18). Research in this area has shown that patients' experiences, understanding as well as quality of SDM was improved when digital PDAs contained visual aids such as icons and bar charts (82, 83). Another helpful feature in this context might be that information is presented in different formats such as writing, video and audio (84, 85). Assessing PDAs for readability and cultural sensitivity of different patient groups can be a strategy for ensuring proper personalization of digital PDAs (85). In terms of interaction with digital PDAs, more research is needed to establish an appropriate framework for personalized design that would also take into consideration emotional aspects of decision-making (86). Another suggestion related to improved interaction with digital PDAs was developing them with a flexible, dynamic design that would enable them to choose questions and topics depending on the patient's individual needs and preferences as opposed to having algorithmic predefined structure and questions (87). This can be particularly helpful for patients with depression that might suffer from cognitive deficits. However, more research is needed to identify the necessary features in this context.

The implementation challenges might be approached by the following strategies. Clinicians need training on how to effectively use PDAs with patients in the SDM process (84, 88). In addition, PDAs can have incorporated communication aids (84) and instruction sheets for their implementation in the clinical workflow (88). Finally, an efficient strategy might be to align PDAs and their development directly with clinical practice guidelines so that the PDAs reflect guideline content and guidelines contain passages on SDM and PDAs (82, 87, 89). This effort might be strengthened by collaborating with initiatives and stakeholders focusing on clinical practice guidelines, SDM and implementation of best available evidence (82, 87). Multistakeholder teams can also help with regular evidence update of information provided in PDAs (90). The quality of evidence in PDAs was identified as an important issue in several included studies in this review (26, 50, 75, 77). Furthermore, there was a strong recommendation to strengthen the evidence in a recent evaluation of Ottawa Decision Support Framework for PDAs (80). Research on formulating guidance for evidence selection and summarization might be particularly helpful in this context (90).

In future studies, it is of importance to further improve the inclusion of preferences, values, and experiences of patients with depression in PDAs and SDM. More scientific studies, particularly qualitative studies, are needed to learn about patients' views, experience, and factors influencing acceptance and implementation of PDAs (91). Furthermore, the topics of personalization and appropriateness of different forms of PDAs for different groups deserve more attention as well as the potential for serious adverse events, such as suicide. Both could be achieved by following user-centered designs by developing PDAs to ensure that important stakeholders (55) and preferences and values of different patient groups are included (27). Gibson et al. (92) suggested giving patients the option to decide how much they want to be involved in the SDM process as this preference might change from patient to patient. It would be also interesting to study the precise role of PDAs in the SDM process; particularly, if there is a change in acceptance and effectiveness depending on the phase in the SDM process in which PDAs are used. Finally, even though privacy was listed as a positive aspect of digital PDAs in our results, more research from an ethical perspective is needed as digitalization in health care raises many challenges such as privacy issues, equality of access, or security. The fact that such ethical issues were not discussed is an important research gap.

### 4.2. Strengths and limitations

Strengths of this review are the systematic literature search with broad inclusion criteria, capturing the full scope of scholarly articles on advantages and disadvantages of PDAs for the treatment of depression. However, this review did not include research on self-help groups, online fora, or patient versions of clinical guidelines, that are probably often used as PDA although not meeting our PDA definition. Also, while we did not exclude articles based on the age of the target group of the respective PDA, due to differences in diagnostic categories between child and adolescent and adult mental health care, our search strategy was likely less sensitive towards articles on child and adolescent depression. The final limitation is that even though we included articles in German, we did not specifically search for articles in German databases.

## 5. Conclusion

The present scoping review suggests that in the field of PDAs for the treatment of patients with depression, more systematic and comprehensive research is needed to study the role of PDAs in the SDM process and to address the potential benefits as well as challenges that digitalized PDAs can offer. More research is also needed regarding effective strategies for the implementation of digital PDAs into the clinical workflow, ethical and equity issues raised by the digital format, and opportunities of tailoring PDAs for diverse patient groups.

## Data Availability

The original contributions presented in the study are included in the article/**Supplementary Material**, further inquiries can be directed to the corresponding author.
