# Supplementary material for: Comparison of analog and digital patient decision aids for the treatment of depression: a scoping review
Source: Front Digit Health. 2023 Sep 1;5:1208889. doi: 10.3389/fdgth.2023.1208889 (PMC10513051; doi:10.3389/fdgth.2023.1208889)
Supplement: Supplementary file 1 [file Table1.docx]

**Supplementary Table 1: Syntax of Search Strategy**

Search strategy for PUBMED

("Decision Support Techniques"[MeSH Terms] OR "decision making, shared"[MeSH Terms] OR "Patient Participation"[MeSH Terms] OR "decision_support"[Title/Abstract] OR "decision aid" [Title/Abstract] OR decision making aid*[Title/Abstract] OR "decision tool"[Title/Abstract] OR "shared decision"[Title/Abstract])

AND

("Mood Disorders"[MeSH Terms] OR "Depressive Disorder"[MeSH Terms] OR "Depression"[MeSH Terms] OR "Antidepressive Agents"[MeSH Terms] OR mood disorder*[Title/Abstract] OR affective disorder*[Title/Abstract] OR depress*[Title/Abstract] OR melanchol*[Title/Abstract])

AND

(english[Filter] OR german[Filter])

NOT

Animal [Filter]

Search strategy for Web of Science

(TS=(decision NEAR/5 (support OR aid OR tool OR shared) OR "patient participation"))

AND

TS=("mood disorder$" OR antidepress* OR "affective disorder$" OR depress* OR melanchol*)

Filters - Language: English, German

Search strategy for PsycINFO

(DE "Decision Support Systems" OR DE "Decision Making" OR DE "Patient Centered Care" OR TI "decision?support" OR AB "decision?support" OR TI "decision aid" OR AB "decision aid" OR TI "decision?making aid*" OR AB "decision?making aid*" OR TI "decision tool" OR AB "decision tool" OR TI "shared decision" OR AB "shared decision")

AND

(DE "Major Depression" OR DE "Endogenous Depression" OR DE "Late Life Depression" OR DE "Postpartum Depression" OR DE "Reactive Depression" OR DE "Recurrent Depression" OR DE "Treatment Resistant Depression" OR DE "Affective Disorders" OR DE "Seasonal Affective Disorder" OR DE "Antidepressant Drugs" OR TI "mood disorder*" OR AB "mood disorder*" OR TI "affective disorder*" OR AB "affective disorder*" OR TI depress* OR AB depress* OR TI melanchol* OR AB melanchol*)

Limiters - Language: English, German; Population Group: Human; Exclude Dissertations
