# Supplementary material for: Comparison of analog and digital patient decision aids for the treatment of depression: a scoping review
Source: Front Digit Health. 2023 Sep 1;5:1208889. doi: 10.3389/fdgth.2023.1208889 (PMC10513051; doi:10.3389/fdgth.2023.1208889)
Supplement: Supplementary file 2 [file Table2.docx]

| Measured Construct | Instrument | Measurement Result | Effect size | Study | Study Design | Type of PDA |
| --- | --- | --- | --- | --- | --- | --- |
| Satisfaction with the PDA | Computer System Usability Questionnaires (CSUQ; 1) | High usability (MED = 6.28, range 4.21-7.00) | n.c. | (2) | Uncontrolled posttest-only study, *n*_patients_ = 10 | digital |
|  | Questionnaire with 5-point Likert scales adapted from (3) | 85.7 % answering neutral/agree/strongly agree | n.c. | (4) | Uncontrolled posttest-only study, *n*_patients_ = 35 | digital |
|  | Unvalidated Likert scales | (strong) agreement on usefulness (91.8%), ease of navigation (88.5%), visual appeal (88.5%), entertainingness (90.1%), clarity of information (88.5%), intention to use (80.3%), intention to recommend (78.8%)  85.2% rated information quantity “too much” | n.c. | (5) | Uncontrolled posttest-only study, *n*_patients_ = 61 | digital |
|  | Unvalidated Acceptability Questionnaire | All responded that the information was neither too much, nor too little, that the aid was neither too long nor too short, and that the information on options was balanced, slanted towards neither option. | n.c. | (6) | Uncontrolled posttest-only study, *n*_patients_ = 5 | digital |
|  | Unvalidated Acceptability Questionnaire with 5-point Likert scales | Overall satisfaction 4.2 ± 0.5 | n.c. | (7) | Uncontrolled posttest-only study, *n*_patients_ = 23 | digital |
|  | Unvalidated Likert scale of helpfulness | 72% rated “helpful” or “very helpful” | n.c. | (8) | Uncontrolled posttest-only study, *n*_patients_ = 49 | digital |
|  | Preparation for Decision-Making Scale (PDMS) patient version (theoretical range 0-100) | 83.8 ± 16.6 | n.c. | (9) | Posttest-only study, *n*_patients_ = 378 | analog |
|  | Preparation for Decision-Making Scale (PDMS) physician version (theoretical range 0-100) | 84.1 ± 12.7 | n.c. | (9) | Posttest-only study, *n*_physicians_ = 374 | analog |
| Patient knowledge on treatment of MDE | Quiz adapted from (3) | Δ_M_ = 0.8 % correct answers, 95% CI [-2.95; 4.54], n.s. | -0.07 | (4) | Randomized, pre-post between-subjects design with active control group, *n*_patients_ = 82 | digital |
|  | Unvalidated quiz with 3 yes/no-questions | 95% correct answers | n.c. | (8) | Uncontrolled posttest-only study, *n*_patients_ = 49 | digital |
|  | Unvalidated 8-item quiz | 6.89 ±1.25 correct answers vs. 4.63 ± 1.48, *p* < .001 | 1.64 | (5) | Randomized, posttest-only study, with TAU control group, *n*_patients_ = 147 | digital |
|  | Unvalidated 7-point Likert scale of subjective knowledge increase | 72.1% rated ≥ 6 | n.c. | (5) | Uncontrolled posttest-only study, *n*_patients_ = 61 | digital |
|  | Unvalidated questionnaire | ANCOVA *b* = 0.1, 95% CI [-0.9; 1.1], n.s. | 0.00 | (7) | Randomized, pre-post between-subjects design with active control group, *n*_patients_ = 46 | digital |
|  | Unvalidated quiz [% correct answers] | PDA 63.5 % ± 21.5 vs. TAU 56.3 % ± 18.4, *p* = .030 | 1.24 | (10) | Stratified randomized posttest only study with TAU control group, *n*_patients_ = 253 | analog |
|  | Depression Decision Quality Instrument (DQI), knowledge score [% correct answers] | PDA: 64 % ± 16 vs. TAU 61 % ± 14, *p* = .041 | 0.20 | (11) | Randomized posttest-only study with TAU control group,  *n*_patients_ = 385 | analog |
| Patient attitudes | Belief about Medicine Questionnaire (BMQ; 12) – necessity scale | after 3 months: PDA: 18.7 ± 3.8 vs. TAU: 18.5 ± 4.2, *t* = 0.5, n.s.  after 6 months: PDA: 19.0 ± 3.8 vs. TAU: 18.5 ± 4.2, *t* = 0.9, n.s. | 0.07  0.13 | (13) | Randomized controlled repeated measures design with TAU control group, *n*_patients_ = 220 | analog |
|  | Belief about Medicine Questionnaire (BMQ) – concerns scale | after 3 months: PDA: 5.8 ± 1.9 vs. TAU: 5.0 ± 2.0, *t* = 1.7, n.s.  after 6 months: PDA: 12.1 ± 3.1 vs. TAU: 13.1 ± 4.0, *t* = 2.3, *p* = .002 | 0.23  -0.31 |  |  |  |
| Decisional Conflict | Decisional Conflict Scale (DCS; 14) | 23.7 ± 15.9 vs. 35.4 ± 15.3, *p* < .001 | -0.75 | (5) | Randomized, posttest-only study, with TAU control group, *n*_patients_ = 147 | digital |
|  |  | 42.2 ± 14.4 vs. 28.1 ± 4.1 | n.c. | (6) | Uncontrolled, pre-post within-subjects design, *n*_patients_ = 5 | digital |
|  |  | Δ_M_ = -17.8, SD = 18.7, *p* < .001 | -0.95 | (15) | Uncontrolled, pre-post within-subjects design, *n*_patients_ = 57 | digital |
|  |  | ANCOVA *b* = -3.5, 95% CI [-12.6; 5.6], n.s. | -0.12 | (7) | Randomized, pre-post between-subjects design with active control group, *n*_patients_ = 46 | digital |
|  |  | Δ_M_ = -7.0, 95% CI [-14.3; 0.3], *p* = .060 | -0.46 | (4) | Randomized, pre-post between-subjects design with active control group, *n*_patients_ = 84 | digital |
|  |  | Δ_M_ = -5.3, 95% CI [-9.5; -1.1], *p* = .010  (reverse coded to align with standard scoring) | -0.16 | (10) | Stratified randomized posttest only study with TAU control group, *n*_patients_ = 252 | analog |
|  | Decisional Conflict Scale (DCS) physician version | Δ_M_ = -11.4, 95% CI [-17.1; -5.7], *p* < .001  (reverse coded to align with standard scoring) | -0.24 | (10) | Stratified randomized posttest only study with TAU control group, *n*_patients_ = 262 | analog |
|  | Combined Outcome Measure for Risk Communication and Treatment Decisionmaking Effectiveness (COMRADE; 16) - subscale confidence in decision | MED = 41 (IQR = 6) vs. MED = 37 (IQR = 7), *p* = .005 | n.c. | (17) | Randomized posttest-only study with TAU control group, *n*_patients_ = 85 | analog |
|  | Patient ability to make a decision (binary item) | Pre: 79% vs. Post: 97%, McNemar’s *p* = .022 | n.c. | (15) | Uncontrolled, pre-post within-subjects design, *n*_patients_ = 57 | digital |
| Patient involvement in decision-making | Shared decision-making questionnaire (SDMQ-11; 18) | High perceived involvement (*M* = 37.4 ± 4.30) | n.c. | (15) | Uncontrolled posttest-only study, *n*_patients_ = 57 | digital |
|  | Shared Decision-Making Questionnaire (SDMQ-9; 19) | PDA: 78.3 ± 18.9 vs. TAU: 80.8 ± 18.1, *z* = −0.259, n.s. | -0.11 | (20) | Posttest-only between-subjects design with TAU control group, *n*_patients_ = 24 | digital |
|  |  | PDA: 85.8 ± 13.8 vs. control: 83.3 ± 13.9, *p* = .026 | 0.18 | (9) | Posttest-only study with active control group (SDM script for physicians plus PDA vs. SDM script for physicians), *n*_patients_ = 378 | analog |
|  | Shared Decision-Making Questionnaire, physician version (SDM-Q-Doc; 21) | PDA: 86.5 ± 9.9 vs. control: 81.1 ± 10.9, *p* < .001 | 0.53 | (9) | Posttest-only study with active control group (SDM script for physicians plus PDA vs. SDM script for physicians), *n*_physicians_ = 374 | analog |
|  | CollaboRATE (22) | PDA: 30.0% giving the highest possible rating vs. TAU: 42.9%, n.s. | -0.31 | (20) | Posttest-only between-subjects design with TAU control group, *n*_patients_ = 24 | digital |
|  | Doctor Facilitation subscale of the Patients’ Perceived Involvement in Care Scale (PICS-DF; 23) | PDA: 15.4 ± 3.5 vs. 17.4 ± 3.1, *p* = .001  TAU: 14.7 ± 3.7 vs. 14.5 ± 3.3, n.s.  Group x time interaction: *p* = .028 | 0.61 | (24) | cluster-randomized, pre-post between-subjects design with TAU control group, *n*_physicians_ = 23, *n*_patients_ = 405 | analog |
|  | Information seeking subscale of the Patients’ Perceived Involvement in Care Scale (PICS-IS; 23) | PDA: 12.3 ± 2.7 vs. 12.3 ± 3.4, n.s.  TAU: 11.3 ± 2.9 vs. 10.3 vs. 2.9, n.s.  Group x time interaction: n.s. | 0.36 | (24) | cluster-randomized pre-post between-subjects design with TAU control group, *n*_physicians_ = 23, *n*_patients_ = 405 | analog |
|  | Man-Son-Hing Scale (MSH; ) | PDA: 26.3 ± 4.0 vs. 28.0 ± 2.9, *p* = .010  TAU: 24.5 ± 3.7 vs. 25.5 ± 3.0, n.s.  Group x time interaction: n.s. | 0.18 | (24) | cluster-randomized pre-post between-subjects design with TAU control group, *n*_physicians_ = 23, *n*_patients_ = 405 | analog |
|  | Combined Outcome Measure for Risk Communication and Treatment Decisionmaking Effectiveness (COMRADE; 16) - subscale satisfaction with communication | MED = 44 (IQR = 9) vs. MED = 38 (IQR = 7), *p* < .001 | n.c. | (17) | Randomized posttest-only study with TAU control group, *n*_patients_ = 85 | analog |
|  | OPTION scale (25) | Δ_M_ = 15.8, 95% CI [6.5; 25.9], *p* = .001 | 0.34 | (10) | Stratified randomized posttest-only study with TAU control group, *n*_patients_ = 96 | analog |
| decision quality | Patient-reported Satisfaction with Decision Scale (SWD; 26) | High satisfaction (*M* = 25.8 ± 3.14) | n.c. | (15) | Uncontrolled posttest-only study, *n*_patients_ = 57 | digital |
|  |  | PDA: 3.1 ± 0.2 vs. TAU: 3.1 ± 0.3, *t*(166) = 0.1, n.s. | 0.01 | (27) | Cluster randomized posttest-only between-subjects study with TAU control group, *n*_patients_ = 168 | analog |
|  | Concordance of decision with patient preferences (binary item) | 100% | n.c. | (15) | Uncontrolled posttest-only study, *n*_patients_ = 53 | digital |
|  | Concordance of decision with patient preferences (researcher-rated) | 37.1% vs. 39.1%, n.s. | -0.05 | (5) | Randomized, with TAU control group, *n*_patients_ = 131 |  |
|  | Concordance of decision with patient needs and values (binary item) | 100% | n.c. | (15) | Uncontrolled posttest-only study, *n*_patients_ = 53 | digital |
|  | Physician-reported Satisfaction with Decision Scale (SWD; 26) | High satisfaction (*M* = 25.4, 95% CI [23.7;27.1]) | n.c. | (15) | Uncontrolled posttest-only study, *n*_physicians_ = 21 | digital |
|  | Concordance of patient decision with guidelines (binary item; researcher-rated) | Pre: 70% vs. Post: 93%, McNemar’s *p* = .004 | n.c. | (15) | Uncontrolled, pre-post within-subjects design, *n*_patients_ = 57 | digital |
| Consultation time | [min] | PDA: 31.4 ± 15.1 vs. 29.2 ± 10.7, n.s.  TAU: 30.9 ± 25.4 vs. 26.7 ±12.5, n.s.  Group x time interaction n.s. | -0.10 | (24) | cluster-randomized pre-post between-subjects design with TAU control group, *n*_physicians_ = 23, *n*_patients_ = 405 | analog |
|  |  | MED = 26 (IQR = 5) vs. MED = 24 (IQR = 22), n.s. | n.c. | (17) | Randomized between-subjects design with TAU control group, *n*_patients_ = 88 | analog |
|  |  | PDA: 15.0 ± 7.0 vs. control: 16.0 ± 7.0, n.s. | -0.14 | (9) | Posttest-only study with active control group (SDM script for physicians plus PDA vs. SDM script for physicians), *n*_physicians_ = 374, *n*_patients_ = 378 | analog |
| Treatment | Defined daily doses (DDD) of prescribed antidepressants | *F* = 7.6, *p* = .001  Pairwise comparisons:  PDA vs. information: Δ_M_ = -18.2 DDD, *p* = .011  no intervention vs. information: Δ_M_ = -33.0 DDD, *p* < .001 | -0.01  -0.02 | (28) | Randomized posttest-only study with two control groups (no intervention and information material), *n*_patients_ = 1190 | digital |
|  | Decision for active treatment (medication or psychotherapy) | PDA: 75.5 % vs. TAU: 52.9 %, χ^2^ (1)= 9.4, *p* = .002 | 0.49 | (27) | Cluster randomized posttest-only between-subjects study with TAU control group, *n*_patients_ = 168 | analog |
| Treatment adherence | Unvalidated 5-point Likert scale | PDA: 4.3 ± 0.8 vs. 4.3 ± 0.9, n.s.  TAU: 3.9 ± 0.8 vs. 3.9 ±1.0, n.s.  Group x time interaction n.s. | 0.00 | (24) | cluster-randomized pre-post between-subjects design with TAU control group, *n*_physicians_ = 23, *n*_patients_ = 405 | analog |
|  | unvalidated 5-point Likert scale (physician-reported) | PDA: 4.3 ± 0.9 vs. 4.8 ± 0.6, *p* = .067  TAU: 4.2 ± 1.1 vs. 4.3 ± 1.1, n.s.  Group x time interaction n.s. | 0.41 | (24) | cluster-randomized pre-post between-subjects design with TAU control group, *n*_physicians_ = 23, *n*_patients_ = 405 | analog |
|  | Unvalidated visual analog scale | after 3 months: MED = 9.0 (IQR = 2.7) vs. MED = 9.3 (IQR = 2.3), n.s.  after 6 months: MED = 9.2 (IQR = 4.9) vs. MED = 8.9 (IQR = 2.3), n.s. | n.c.  n.c. | (17) | Randomized posttest-only study with TAU control group, *n*_patients_ = 66 | analog |
|  | Primary adherence (according to pharmacy record)  Secondary adherence (according to pharmacy record) | PDA: 93.2 % vs. TAU: 86.2 %, *p* = .19  PDA: 97.7 % vs. TAU: 98.0 %, *p* = .25 | n.c.  n.c. | (10) | Stratified randomized posttest-only study with TAU control group, *n*_patients_ = 206 | analog |
|  | Proportion of antidepressant pills taken and psychotherapy sessions attended | PDA: 0.18 ± 0.03 vs. TAU: 0.10 ± 0.03, *z* = 1.77, n.s. | 1.4 | (27) | Cluster randomized repeated measures between-subjects study with TAU control group, *n*_patients_ = 181 | analog |
|  | Morisky Medication Adherence Scale (MMAS; 29) | after 3 months: PDA: 5.8 ± 1.9 vs. TAU: 5.0 ± 2.0, *t* = 2.8, *p* = .004  after 6 months: PDA: 6.0 ± 1.9 vs. TAU: 4.9 ± 1.9, *t* = 4.1, *p* < .001 | 0.39  0.55 | (13) | Randomized controlled repeated measures design with TAU control group, *n*_patients_ = 220 | analog |
| Treatment outcome | Patient Health Questionnaire (PHQ; 30) – short form | PDA: 35.5 ± 49.6 % reduction vs. 50.6 ± 35.3, n.s.  TAU: 12.4 ± 47.8 vs. 45.9 ± 34.2, n.s.  Group x time interaction n.s. | -0.38 | (24) | cluster-randomized pre-post between-subjects design with TAU control group, *n*_physicians_ = 23, *n*_patients_ = 405 | analog |
|  |  | after 3 months: Δ_M_ = 0.4, 95% CI [-2.5; 3.4], n.s.  after 6 months: Δ_M_ = -0.2, 95% CI [-2.9; 2.6], n,s, | 0.02  -0.01 | (10) | Stratified randomized posttest-only study with TAU control group, *n*_patients_ = 215  *n*_patients_ = 210 | analog |
|  | Quick Inventory of Depressive Symptomatology Self Report (QIDS-SR; 31) | after 3 months: MED = 10 (IQR = 6) vs. MED = 10 (IQR = 8), n.s.  after 6 months: MED = 10 (IQR = 9) vs. MED = 10 (IQR = 9), n.s. | n.c.  n.c. | (17) | Randomized posttest-only study with TAU control group, *n*_patients_ = 88 | analog |
|  | Hamilton Depression Rating Scale (HAM-D; 32) | Treatment x time interaction: *F*(3, 580) = 0.75, n.s. | n.c. | (27) | Cluster randomized repeated measures between-subjects study with TAU control group, *n*_patients_ = 202 | analog |
|  | Montgomery–Åsberg Depression Scale (MADRS; 33) | after 3 months: PDA: 21.1 ± 12.2 vs. TAU: 21.0 ± 12.6, *t* = 0.04, n.s.  after 6 months: PDA: 20.7 ± 12.0 vs. TAU: 20.9 ± 12.5, *t* = 0.1, n.s. | 0.01  -0.01 | (13) | Randomized controlled repeated measures design with TAU control group, *n*_patients_ = 220 | analog |
|  | Edinburgh Postnatal Depression Scale (EPDS; 34) | PDA: 14.5 ± 7.1 vs. 12.8 ± 6.6  Control: 12.5 ± 6.2 vs. 10.3 ± 5.9  ANCOVA *b* = 1.4, 95% CI [-1.6; 4.5], n.s. | 0.07 | (7) | Randomized, pre-post between-subjects design with active control group, *n*_patients_ = 46, t_0_ 🡪 t_1_ (after four weeks) | digital |
|  |  | PDA: 14.5 ± 7.1 vs. 9.0 ± 4.8  Control: 12.5 ± 6.2 vs. 9.7 ± 5.5  ANCOVA *b* = -1.3, 95% CI [-4.1; 1.5], n.s. | -0.40 |  | Randomized, pre-post between-subjects design with active control group, *n*_patients_ = 44, t_0_ 🡪 t_2_ (12 weeks postpartum or 6 months after t_0_) | digital |
|  |  | PDA: 12.3 ± 5.5 vs. 9.0 ± 5.2  Control: 11.9 ± 4.5 vs. 10.3 ± 4.9  Δ_M_ = -1.5, 95% CI [-3.4; 0.5], n.s. | -0.39 | (4) | Randomized, pre-post between-subjects design with active control group, *n*_patients_ = 84 | digital |
|  | State-Trait Anxiety Inventory (STAI; 35) | PDA: 44.0 ± 15.2 vs. 44.8 ± 15.5  Control: 41.8 ± 12.8 vs. 38.1 ± 12.3  ANCOVA *b* = 5.1, 95% CI [1.0; 11.3], n.s. | 0.32 | (7) | Randomized, pre-post between-subjects design with active control group, *n*_patients_ = 46, t_0_ 🡪 t_1_ (after four weeks) | digital |
|  |  | PDA: 44.0 ± 15.2 vs. 38.8 ± 13.7  Control: 41.8 ± 12.8 vs. 42.2 ± 12.6  ANCOVA *b* = -3.8, 95% CI [-11.4; 3.8], n.s. | -0.39 | (7) | Randomized, pre-post between-subjects design with active control group, *n*_patients_ = 43, t_0_ 🡪 t_2_ (12 weeks postpartum or 6 months after t_0_) | digital |
|  |  | PDA: 43.6 ± 11.7 vs. 39.8 ± 14.5  Control: 42.8 ± 13.0 vs. 42.2 ± 13.3  Δ_M_ = -2.6, 95% CI [-7.9; 2.7], n.s. | -0.26 | (4) | Randomized, pre-post between-subjects design with active control group, *n*_patients_ = 84 | digital |
|  | sick leave days | *F* = 0.03, n.s.  PDA vs. information:  no intervention vs. information: | -0.00 -0.01 | (28) | Randomized, posttest-only study with two control groups (no intervention and information material), *n*_patients_ = 1190 | digital |
|  | European Quality of Life 5 Dimensions (EQ-5D; 36) | after 3 months: PDA: 0.7 ± 0.4 vs. TAU: 0.7 ± 0.4, *t* = 0.08, n.s.  after 6 months: PDA: 0.7 ± 0.4 vs. TAU: 0.7 ± 0.4, *t* = 0.36, n.s. | 0.01  0.05 | (13) | Randomized controlled repeated measures design with TAU control group, *n*_patients_ = 220 | analog |
|  | inpatient treatment days | *F* = 0.4, n.s.  PDA vs. information: no intervention vs. information: | 0.01 -0.07 | (28) | Randomized, posttest-only study with two control groups (no intervention and information material), *n*_patients_ = 1190 | digital |
| Satisfaction with care | Clients Satisfaction Questionnaire (CSQ-8; 37) | 29.8 ± 2.7 vs. 27.0 ± 3.6, *p* = .014 | 0.38 | (24) | cluster-randomized between-subjects design with TAU control group, *n*_physicians_ = 23, *n*_patients_ = 194 | analog |
|  |  | MED = 24 (IQR = 2) vs. MED = 24 (IQR = 3), n.s. | n.c. | (17) | Randomized posttest-only study with TAU control group, *n*_patients_ = 85 | analog |
|  | Treatment Satisfaction Questionnaire for Medication (TSQM; 38) | after 3 months: PDA: 86.7 ± 11.1 vs. TAU: 82.8 ± 13.4, *t* = 2.33, *p* = .021  after 6 months: PDA: 88.7 ± 10.8 vs. TAU: 82.9 ± 13.4, *t* = 3.55, *p* < .001 | 0.31  0.48 | (13) | Randomized controlled repeated measures design with TAU control group, *n*_patients_ = 220 | analog |
| *Notes*. Shown are quantitative results on the effect of patient decision aids (PDA), not shown are quantitative results of complex interventions including but going beyond PDA. The direction of the effect size being arbitrary (32), all *d*s are presented as positive when the PDA resulted in numerically greater outcomes and *vice versa*. Measures are patient-reported unless stated otherwise. MED = median, TAU = treatment as usual, SDM = shared decision-making, CI = confidence interval, IQR = interquartile range, n.c. = not computable, n.s. = not significant | | | | | | |

**Supplementary Table 2:** Overview of quantitative results.

REFERENCES

1. Lewis JR. Computer system usability questionnaire. *International Journal of Human-Computer Interaction* (1995).

2. Dannenberg MD, Bienvenida JC, Bruce ML, Nguyen T, Hinn M, Matthews J, et al. End-user views of an electronic encounter decision aid linked to routine depression screening. *Patient Educ Couns* (2019) **102**:555–63. doi:10.1016/j.pec.2018.10.002

3. Metcalfe KA, Poll A, O’connor A, Gershman S, Armel S, Finch A, et al. Development and testing of a decision aid for breast cancer prevention for women with a BRCA1 or BRCA2 mutation. *Clin Genet* (2007) **72**:208–17.

4. Vigod SN, Hussain-Shamsy N, Stewart DE, Grigoriadis S, Metcalfe K, Oberlander TF, et al. A patient decision aid for antidepressant use in pregnancy: Pilot randomized controlled trial. *J Affect Disord* (2019) **251**:91–9. doi:10.1016/j.jad.2019.01.051

5. Perestelo-Perez L, Rivero-Santana A, Sanchez-Afonso JA, Perez-Ramos J, Castellano-Fuentes CL, Sepucha K, et al. Effectiveness of a decision aid for patients with depression: A randomized controlled trial. *Health Expect* (2017) **20**:1096–105. doi:10.1111/hex.12553

6. Shillington AC, Langenecker SA, Shelton RC, Foxworth P, Allen L, Rhodes M, et al. Development of a patient decision aid for treatment resistant depression. *J Affect Disord* (2020) **275**:299–306. doi:10.1016/j.jad.2020.07.014

7. Khalifeh H, Molyneaux E, Brauer R, Vigod S, Howard LM. Patient decision aids for antidepressant use in pregnancy: a pilot randomised controlled trial in the UK. *BJGP Open* (2019) **3**. doi:10.3399/bjgpopen19X101666

8. Reis J. A Case Report of College Students' Rating of a Shared Decision-Making Tool for Taking Antidepression Medication. *J Patient Exp* (2021) **8**:23743735211007352. doi:10.1177/23743735211007352

9. Abousheishaa AA, Lazim NH, Tang SL, Sulaiman AH, Huri HZ, Guan NC. Antidepressant decision aid for major depressive disorder patients (ADAM): Development and pilot testing. *Patient Educ Couns* (2022) **105**:2466–74. doi:10.1016/j.pec.2021.11.007

10. LeBlanc A, Herrin J, Williams MD, Inselman JW, Branda ME, Shah ND, et al. Shared Decision Making for Antidepressants in Primary Care: A Cluster Randomized Trial. *JAMA Intern Med* (2015) **175**:1761–70. doi:10.1001/jamainternmed.2015.5214

11. Brodney S, Valentine KD, Sepucha K. Psychometric evaluation of a decision quality instrument for medication decisions for treatment of depression symptoms. *BMC Med Inform Decis Mak* (2021) **21**:252. doi:10.1186/s12911-021-01611-w

12. Horne R, Weinman J, Hankins M. The beliefs about medicines questionnaire: the development and evaluation of a new method for assessing the cognitive representation of medication. *Psychol Health* (1999) **14**:1–24.

13. Aljumah K, Hassali MA. Impact of pharmacist intervention on adherence and measurable patient outcomes among depressed patients: a randomised controlled study. *BMC Psychiatry* (2015) **15**:219. doi:10.1186/s12888-015-0605-8

14. Garvelink MM, Boland L, Klein K, Nguyen DV, Menear M, Bekker HL, et al. Decisional conflict scale use over 20 years: the anniversary review. *Med Decis Making* (2019) **39**:301–14.

15. Simmons MB, Elmes A, McKenzie JE, Trevena L, Hetrick SE. Right choice, right time: Evaluation of an online decision aid for youth depression. *Health Expect* (2017) **20**:714–23. doi:10.1111/hex.12510

16. Edwards A, Elwyn G, Hood K, Robling M, Atwell C, Holmes-Rovner M, et al. The development of COMRADE—a patient-based outcome measure to evaluate the effectiveness of risk communication and treatment decision making in consultations. *Patient Educ Couns* (2003) **50**:311–22.

17. Aoki Y, Takaesu Y, Inoue M, Furuno T, Kobayashi Y, Chiba H, et al. Seven-day shared decision making for outpatients with first episode of mood disorders among university students: A randomized controlled trial. *Psychiatry Res* (2019) **281**:112531. doi:10.1016/j.psychres.2019.112531

18. Simon D, Schorr G, Wirtz M, Vodermaier A, Caspari C, Neuner B, et al. Development and first validation of the shared decision-making questionnaire (SDM-Q). *Patient Educ Couns* (2006) **63**:319–27.

19. Kriston L, Scholl I, Hölzel L, Simon D, Loh A, Härter M. The 9-item Shared Decision Making Questionnaire (SDM-Q-9). Development and psychometric properties in a primary care sample. *Patient Educ Couns* (2010) **80**:94–9.

20. Barr PJ, Forcino RC, Dannenberg MD, Mishra M, Turner E, Zisman-Ilani Y, et al. Healthcare Options for People Experiencing Depression (HOPE*D): the development and pilot testing of an encounter-based decision aid for use in primary care. *BMJ Open* (2019) **9**:e025375. doi:10.1136/bmjopen-2018-025375

21. Scholl I, Kriston L, Dirmaier J, Buchholz A, Härter M. Development and psychometric properties of the Shared Decision Making Questionnaire–physician version (SDM-Q-Doc). *Patient Educ Couns* (2012) **88**:284–90.

22. Barr PJ, Thompson R, Walsh T, Grande SW, Ozanne EM, Elwyn G. The psychometric properties of CollaboRATE: a fast and frugal patient-reported measure of the shared decision-making process. *J Med Internet Res* (2014) **16**:e3085.

23. Lerman CE, Brody DS, Caputo GC, Smith DG, Lazaro CG, Wolfson HG. Patients' Perceived Involvement in Care Scale: relationship to attitudes about illness and medical care. *J Gen Intern Med* (1990) **5**:29–33. doi:10.1007/BF02602306

24. Loh A, Simon D, Wills CE, Kriston L, Niebling W, Härter M. The effects of a shared decision-making intervention in primary care of depression: a cluster-randomized controlled trial. *Patient Educ Couns* (2007) **67**:324–32. doi:10.1016/j.pec.2007.03.023

25. Elwyn G, Hutchings H, Edwards A, Rapport F, Wensing M, Cheung W-Y, et al. The OPTION scale: measuring the extent that clinicians involve patients in decision‐making tasks. *HEALTH EXPECTATIONS* (2005) **8**:34–42.

26. Wills CE, Holmes-Rovner M. Preliminary validation of the Satisfaction With Decision scale with depressed primary care patients. *Health Expect* (2003) **6**:149–59. doi:10.1046/j.1369-6513.2003.00220.x

27. Raue PJ, Schulberg HC, Bruce ML, Banerjee S, Artis A, Espejo M, et al. Effectiveness of Shared Decision-Making for Elderly Depressed Minority Primary Care Patients. *Am J Geriatr Psychiatry* (2019) **27**:883–93. doi:10.1016/j.jagp.2019.02.016

28. Weiss F, Vietor C, Hecke TL. Use of routine data for evaluation purposes in sickness funds - Evaluation of the "TK-Patientendialog". *Gesundheitswesen* (2010) **72**:371–8. doi:10.1055/s-0030-1249702

29. Morisky DE, Green LW, Levine DM. Concurrent and predictive validity of a self-reported measure of medication adherence. *Med Care* (1986):67–74.

30. Spitzer RL, Kroenke K, Williams JB, Patient Health Questionnaire Primary Care Study Group. Validation and utility of a self-report version of PRIME-MD: the PHQ primary care study. *JAMA* (1999) **282**:1737–44.

31. Rush AJ, Trivedi MH, Ibrahim HM, Carmody TJ, Arnow B, Klein DN, et al. The 16-Item Quick Inventory of Depressive Symptomatology (QIDS), clinician rating (QIDS-C), and self-report (QIDS-SR): a psychometric evaluation in patients with chronic major depression. *Biological Psychiatry* (2003) **54**:573–83.

32. Hamilton M. A rating scale for depression. *J Neurol Neurosurg Psychiatry* (1960) **23**:56.

33. Montgomery SA, Åsberg M. A new depression scale designed to be sensitive to change. *BRITISH JOURNAL OF PSYCHIATRY* (1979) **134**:382–9.

34. Gibson J, McKenzie‐McHarg K, Shakespeare J, Price J, Gray R. A systematic review of studies validating the Edinburgh Postnatal Depression Scale in antepartum and postpartum women. *ACTA PSYCHIATRICA SCANDINAVICA* (2009) **119**:350–64.

35. Dennis C-L, Coghlan M, Vigod S. Can we identify mothers at-risk for postpartum anxiety in the immediate postpartum period using the State-Trait Anxiety Inventory? *J Affect Disord* (2013) **150**:1217–20.

36. Group, The EuroQol. EuroQol-a new facility for the measurement of health-related quality of life. *HEALTH POLICY* (1990) **16**:199–208.

37. Schmidt J, Nübling R. “ZUF-8. Fragebogen zur Messung der Patientenzufriedenheit,”. In: Brähler E, Schumacher J, Strauß B, editors. *Diagnostische verfahren in der psychotherapie*. Hogrefe, Verlag für Psychologie (2002). p. 392–6.

38. Atkinson MJ, Sinha A, Hass SL, Colman SS, Kumar RN, Brod M, et al. Validation of a general measure of treatment satisfaction, the Treatment Satisfaction Questionnaire for Medication (TSQM), using a national panel study of chronic disease. *Health Qual Life Outcomes* (2004) **2**:1–13.
